# Myelofibrosis in 2019: moving beyond JAK2 inhibition

**DOI:** 10.1038/s41408-019-0236-2

**Published:** 2019-09-11

**Authors:** Michael Schieber, John D. Crispino, Brady Stein

**Affiliations:** Robert H. Lurie Comprehensive Cancer Center, Division of Hematology/Oncology, Department of Medicine, Feinberg School of Medicine, Northwestern University, Chicago, IL USA

**Keywords:** Myeloproliferative disease, Myeloproliferative disease

## Abstract

Myelofibrosis (MF) is a myeloproliferative neoplasm characterized by ineffective clonal hematopoiesis, splenomegaly, bone marrow fibrosis, and the propensity for transformation to acute myeloid leukemia. The discovery of mutations in *JAK2*, *CALR*, and *MPL* have uncovered activated JAK-STAT signaling as a primary driver of MF, supporting a rationale for JAK inhibition. However, JAK inhibition alone is insufficient for long-term remission and offers modest, if any, disease-modifying effects. Given this, there is great interest in identifying mechanisms that cooperate with JAK-STAT signaling to predict disease progression and rationally guide the development of novel therapies. This review outlines the latest discoveries in the biology of MF, discusses current clinical management of patients with MF, and summarizes the ongoing clinical trials that hope to change the landscape of MF treatment.

## Introduction

Myelofibrosis (MF), including primary MF (PMF), post-essential thrombocythemia MF (post-ET/MF), and post-polycythemia MF (post-PV/MF), is a progressive myeloid neoplasm characterized by clonal ineffective hematopoiesis, extramedullary hematopoiesis, a reactive bone marrow environment resulting in reticulin deposition and fibrosis, and a propensity toward leukemic transformation^1^. Compared with ET, PV, and a novel pathological category termed prefibrotic/early PMF^2^, MF carries the poorest prognosis.

The identification of driver mutations in *JAK2*, *CALR*, and *MPL* has contributed to a better understanding of disease pathogenesis, implicating near-universal upregulation of JAK-STAT signaling, and has led to the development of the sole targeted therapy for MF, the JAK2 inhibitor ruxolitinib. Although this drug has contributed to relief from inflammatory symptoms and splenomegaly, it does not significantly modify the natural history of the disease^3,4^. More recently, the molecular landscape of MF has become increasingly well characterized, leading to the development of genetically based prognostic scoring systems (MIPPS70, MIPSS70+ version 2.0, and GIPPS)^5–7^. These aim to identify higher-risk patients who might benefit from earlier aggressive therapies such as allogeneic stem transplantation (ASCT). A better understanding of the molecular pathogenesis will also foster development of rational therapies, with the aim of modifying the natural history of the disease. Herein, we review the current understanding of the molecular basis of MF and the repertoire of potential new therapies.

## Molecular landscape of PMFand transformation

### Activation of JAK-STAT signaling drives MF

**Fig. 1:**
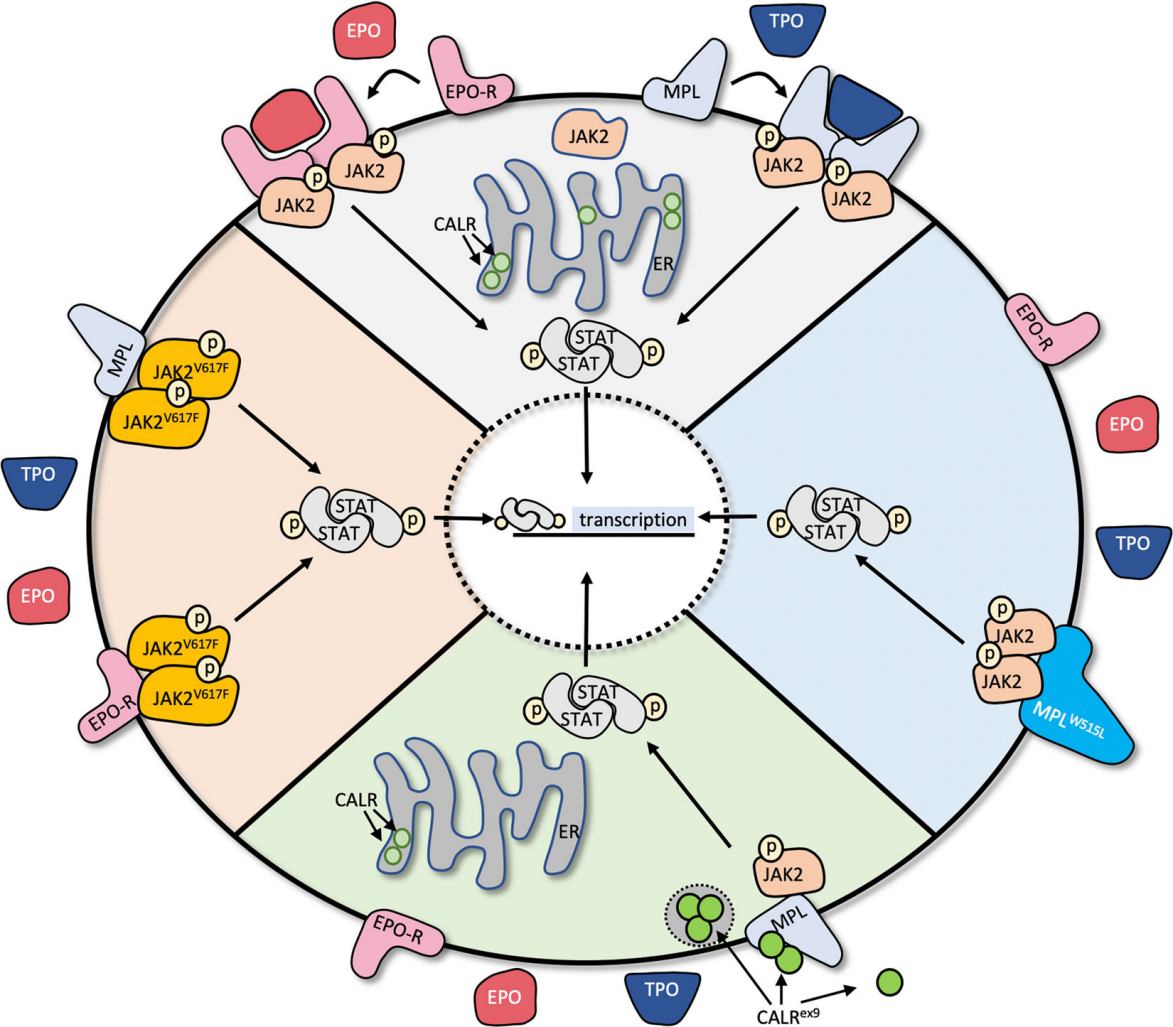
Activated JAK-STAT signaling drives myelofibrosis. In normal physiology (top), binding of erythropoietin (EPO) or thrombopoietin (TPO) to their respective receptors (EPO-R, MPL) leads to phosphorylation and activation of JAK2 resulting in STAT-dependent transcription of target genes. The JAK^V617F^ (left), MPL^W515L^ (right), and CALR exon 9 (CALR^ex9^, bottom) mutations result in constitutive JAK-STAT activation

A central role for JAK/STAT signaling in the pathogenesis of the myeloproliferative neoplasms (MPNs) was discovered by identifying the somatically acquired JAK2^V617F^ mutation in more than 95% of patients with PV and over 50% of patients with MF and ET^8^. JAK2^V617F^ disrupts the autoinhibitory JH2 pseudokinase domain, leading to constitutive activation of JAK2 kinase activity and STAT-mediated activation of transcription (Fig. 1, left). The JAK2^V617F^ mutation alone is sufficient to produce a PV-like phenotype in mouse models, as transplantation of murine JAK2^V617F^ expanding cells into wild-type animals induces trilineage hyperplasia and, with variable penetrance, reticulin fibers in the bone marrow, consistent with early MF^9^. Lower expression levels of JAK2^V617F^ produce a phenotype more consistent with ET, a trend also observed in human patients^10^.

Despite the near-complete incidence of JAK2^V617F^ in PV, a substantial number of patients with ET and MF are JAK2^V617F^ negative, which prompted the investigation into other JAK-STAT signaling transduction pathway members for disease-causing mutations. Exome sequencing of 45 JAK2^V617F^-negative MF patients identified a somatic mutation in the transmembrane of the upstream thrombopoietin (TPO) receptor (MPL^W515L^) in 4 patients^11^. MPL^W515L^ results in constitutive activation of TPO-receptor signaling, JAK2 phosphorylation, and activation of STAT-dependent transcription (Fig. 1, right). Transplantation of murine MPL^W515L^ into irradiated wild-type mice produces a fully penetrant MPN with marked thrombocytosis and increased bone marrow reticulin deposits that correlates with JAK-STAT activation^11^. Larger cohorts have now been evaluated with MPNs and identified *MPL* mutations (MPL^W515L^ as well as MPL^W515K^) in ~5% of cases confirming that, although pathologic, these alterations are only present in a small fraction of ET and MF patients^1^.

The next major breakthrough in understanding the molecular basis of MF came in 2013, when two groups independently identified mutations in exon 9 of calreticulin (*CALR*^*ex9*^) in the majority of *JAK2*- and *MPL*-negative patients with ET and MF^12,13^. CALR is a calcium-binding protein present in the endoplasmic reticulum (ER), where it functions as a protein chaperone and maintains calcium homeostasis. Interestingly, all *CALR* mutations identified were somatic insertions and deletions that produced in a +1 reading frameshift and resulted in a novel C-terminal sequence lacking the ER-targeting KDEL sequence. Over 80% of these frameshift mutations fall into two categories: type 1 (52 bp deletion in exon 9) and type 2 (5 bp insertion within exon 9).

The oncogenic mechanism of the mutant CALR^ex9^ protein is still under investigation. Mutant CALR^ex9^ induces constitutive phosphorylation of JAK2 and activation of STAT transcription in a MPL-dependent manner, as *c-mpl-*deficient mice are protected against the *CALR*^ex9^ disease phenotype. Supporting these observations, patients with CALR-mutated MF respond to JAK2 inhibition^14^. A recently presented study by Pecquet et al.^15^ illustrated that mutant CALR is secreted extracellularly and is detectable in the serum of patients with *CALR*^ex9^ MPNs. This mutant CALR protein may function as a “rogue cytokine” to further enhance TPO-receptor signaling in both autocrine and paracrine fashions^15^. Another notable observation made by these authors is that this novel mechanism of enhanced TPO-signaling is more prominent in CALR-mutated cells than in wild-type counterparts.

### An abnormal epigenetic landscape facilitates clonal selection in MF

With increased sequencing efforts and improved technology, mutations beyond *JAK2*, *MPL*, and *CALR*, which regulate the epigenetic landscape of hematopoietic stem cells (HSCs), have now been identified in patients with MF^16^. For example, recurrent alterations in the 10–11 translocation-2 (*TET2*) DNA dioxygenase are found in 10% of MF patients. TET2 catalyzes the conversion of 5-methylcytosine to 5-hydroxymethylcytosine, an intermediate step towards promoter hypomethylation and transcriptional activation of target genes that promote stem cell differentiation^17^. Hematopoietic-specific deletion of *Tet2*, akin to the somatic mutations accumulated in MF patients, leads to global promoter hypermethylation, increased HSC survival, and propensity towards transformation in mice^18^. Interestingly, *TET2* mutations are mutually exclusive with mutations in the isocitrate dehydrogenase enzymes IDH1 and IDH2^19,20^. This observation led to the discovery that loss of IDH1 and IDH2 results in accumulation of the oncometabolite 2-hydroxyglutarate, inhibiting TET2 activity. *IDH* mutations in MF patients portends a poor prognosis and increased leukemic transformation potential^21^.

Serial transplantation assays of murine *Jak2*^*V617F*^ HSCs show that activated JAK-STAT signaling alone, while sufficient in producing an MPN phenotype, results in premature stem cell exhaustion on secondary transplantation. However, placing the *Jak2*^*V617F*^ mutation in a *Tet2*-null (*Tet2*^*−/−*^) background produces a more severe MPN phenotype, suggesting a clonal advantage of *Jak2*^*V617F*^/*Tet2*^*−/−*^ double mutants^22,23^. Supporting this, *Jak2*^*V617F*^/*Tet2*^*−/−*^ HSCs have a proliferative advantage compared with *Jak2*^*V617F*^/*Tet2*^*WT*^ HSCs and also can be successfully serially transplanted into secondary recipients. Despite this persistence, *Jak2*^*V617F*^/*Tet2*^*−/−*^ mice do not display increased bone marrow fibrosis or leukemic transformation. This phenotype is similar to chronic phase MF in humans in which *TET2* mutations associate with increased age and the presence of JAK2^V617F^, but do not predict disease severity or survival. In addition, there is not yet convincing evidence that restoration of TET2 activity has disease-modifying effects in MF.

Loss or deletions within the long-arm of chromosome 7 impart a poor prognosis in a variety of myeloid disorders. A candidate sequencing approach of genes localized to this region revealed recurrent mutations in *Enhancer of Zeste Homolog 2* (*EZH2*) in 13% of patients with MF^24^. *EZH2* encodes a histone H3 lysine 27 (H3K27) methyltransferase and is the catalytic subunit of the polycomb repressive complex (PRC2), which is required for the maintenance of HSCs^25^. Alterations in *EZH2* are predominately frameshift mutations resulting in truncated protein isoforms or missense mutations within the highly conserved methyltransferase domain, suggesting a tumor suppressor function of *EZH2*. Patients with MF harboring *EZH2* abnormalities have a reduced leukemia-free and overall survival (OS) that is independent of the dynamic international prognostic scoring system (DIPSS) risk category and *JAK2*^*V617F*^ allele burden^26^.

In contrast to *Tet2*, conditional murine knockout of *Ezh2* (*Ezh2*^*−/−*^) in a *Jak2*^*V617F*^ background induces a rapid progression to MF^27,28^. Confirming a cell autonomous mechanism, bone marrow transplantation assays of *Jak2*^*V617F*^/*Ezh2*^*−/−*^ cells leads to a more rapid myelofibrotic phenotype in secondary recipients than in primary donors. Whereas *Jak2*^*V617F*^ animals exhibit at PV-like disease, *Jak2*^*V617F*^/*Ezh2*^*−/−*^ mice display decreased erythroid differentiation and an expansion of megakaryocytic precursors that correlates with peripheral blood thrombocytosis. Given that *EZH2* encodes a H3K27 methyltransferase, loss of *EZH2* function is expected to reduce H3K27me3 levels leading to transcriptional activation of target genes. These include two proinflammatory cytokine-like mediators, S100a8 and S100a9, as well as high-mobility AT-hook 2 (HMGA2). The S100a8, S100a9, and HMGA2 loci display decreased H3K27 trimethylation in *Jak2*^*V617F*^/*Ezh2*^*−/−*^ double mutant HSCs, which correlates with their increased expression. Upregulated HMGA2 expression is observed in human patients with MF compared with PV and ET controls, and correlates with worsened splenomegaly^29^. Thus, loss of *EZH2* transcriptional repression concurrently with activated JAK-STAT results in a more severe MPN phenotype than JAK2^V617F^ alone and favors progression to MF.

Targeted sequencing of MF patients has also identified recurrent mutations in *Addition of Sex Combs Like 1* (*ASXL1*) in at least 10–20% of MF patients, but possibly a higher proportion of triple-negative MF cases^8^. Similar to *EZH2*, loss of *ASXL1* impairs the PRC2-mediated suppression of leukemia oncogenes in hematopoietic progenitors^30^. Conditional deletion of *Asxl1* in murine hematopoietic progenitors resulted in progressive anemia and leukemia with multilineage dysplasia similar to human myelodysplastic syndrome (MDS), but without evidence of MF^31^. Despite these observations in the murine model, mutations in *ASXL1* are clinically relevant in human MF. *ASXL1* mutations negates the favorable risk of *CALR* mutations and its presence alone is associated with poor prognosis^1^.

A key unanswered question is whether epigenetic mutations are clinically targetable. As discussed, *EZH2* and *ASXL1* abnormalities result in inhibition of the PRC2 complex, which reduces H3K27 trimethylation and in turn promotes H3K27 acetylation, ultimately resulting in the recruitment of bromodomain and extraterminal family proteins to gene promotors, to activate transcription^32^. In preclinical murine models, the bromodomain inhibitor JQ1 preferentially reduces bone marrow fibrosis- and MF-initiating clones in the Jak2^V617F^/*Ezh2*^*−/−*^ background^33^. A phase I/II clinical trial is currently underway testing the bromodomain inhibitor CPI-0610 in combination with ruxolitinib in patients with MF (Table 3). Histone deacetylase inhibitors, which are used to promote proper promoter methylation, demonstrated efficacy in murine MF models but enthusiasm for this class in patients has been tempered^34^.

### Abnormal mRNA splicing is present in MF and other myeloid malignancies

Mutations in components of the RNA spliceosome machinery, such as *SRSF2*, *SF3B1*, and *U2AF1*, have been identified in patients with MF as well as a number of other myeloid malignancies^16,35^. In murine models, loss of *Srsf2* produces a hypocellular bone marrow failure phenotype, whereas heterozygous mice with the *Srsf2*^P95H^ mutant have a hypercellular phenotype with myelodysplasia akin to typical MDS^36^. In contrast to the global exon exclusion that occurs with complete loss of SRSF2, *Srsf2*^P95H^ mice exhibit sequence-specific alterations in mRNA splicing. One such alternative splicing event was discovered in *EZH2*, leading to nonsense-mediated decay of the critical epigenetic regulator. Fittingly, *EZH2* and *SRSF2* mutations are mutually exclusive in patients with MDS^37^.

It remains unclear why some patients with spliceosome mutations preferentially develop myelodysplasia rather than MF, but perhaps the degree of JAK-STAT activation plays a role. Furthermore, proper mRNA splicing is a ubiquitous process in all cell types, raising the question whether spliceosome inhibitors can be safely tolerated. In preclinical xenograft models for acute myeloid leukemia (AML); however, the spliceosome inhibitor E7107 preferentially led to intron retention in *Srsf2*-mutated samples and reductions in leukemia burden, suggesting a possible therapeutic window for these agents^38^.

### Mechanisms of post-MPN leukemic transformation

Despite the evidence that loss-of-function mutations in key epigenetic regulators and spliceosome components within HSCs accelerate MF progression and are seen in de novo AML, none predict leukemic transformation alone with high probability. Furthermore, it has been observed that nearly half of the patients who develop AML from prior JAK2^V617F^ MPN are in fact wild type for *JAK2* in their leukemic blasts^39^. Similarly, *TET2* mutations can accumulate in both pre-JAK2^V617F^ and post-JAK2^V617F^ HSC clones^32^. This suggests that, in at least a subset of patients, clonal advantage imparted by enhanced JAK-STAT signaling and associated epigenetic abnormalities are secondary events from an antecedent pre-JAK2^V617F^ HSC clone and not necessarily drivers of transformation.

Sequencing of a number of genes implicated in myeloid malignancies has been performed in samples from patients with post-MPN AML vs. chronic MPN phase. Although present at low levels in chronic phase, a higher variant allele frequency of *TP53* mutations has been observed in post-MPN AML samples, suggesting loss of the wild-type *TP53* allele contributes to clonal expansion^40,41^. Consistent with a role for *TP53* mutations in progression, expression of JAK2^V617F^ in *Tp53*-null mice produced a transplantable leukemia that was sensitive to ruxolitinib^40,41^. Highlighting that there is no uniform path to leukemic transformation, whole genome sequencing of a single patient with post-MF AML demonstrated a *JAK2*-mutated dominant founder clone in chronic phase, a *JAK2*/*ASXL1*/*RUNX1*/*IDH1*-mutated subclone upon AML transformation, and a *JAK2*/*ASXL1*-mutated disease at relapse. No *TP53* mutations were observed in this single case^42^. Therefore, there is significant overlap between known genetic abnormalities in AML and the mutational landscape in MF that predicts transformation. However, no clear temporal sequence or combination of molecular abnormalities is alone sufficient for post-MF leukemia development.

## Genetic-based prognostication in MF

Given the importance of both driver and secondary mutations in patient outcomes, older clinically based prognostic scoring systems, such as the DIPSS, have appropriately evolved. The mutation-enhanced international prognostic scoring system 70+ version 2.0 (MIPSS70+ V2.0), derived from MIPSS, and the genetically inspired prognostic scoring system (GIPPS) are the most recently developed and are applicable in current practice (see Table 1)^6,7^. The MIPSS70+ V2.0 is a five-tiered model developed using an Italian and Mayo Clinic patient cohort, and compared with its predecessors, the MIPSS70 and MIPSS70+ relies less on clinical variables and does not risk stratify based on the presence of bone marrow fibrosis. In its most current form, only anemia, peripheral blast count, and constitutional symptoms impact risk score. Compared with these clinical variables, hazard ratios are much less favorable for high-risk molecular and cytogenetic abnormalities, and appropriately carry more weight in determining risk category. The second version also incorporates the poor-risk *U2AF1* mutation.

**Table 1:** Comparison of genetic-based risk models in myelofibrosis

Clinical or genetic variable	MIPSS70	MIPSS70+V2.0	GIPPS
Anemia	X	X^a^	
Leukocytosis	X		
Thrombocytopenia	X		
Blasts	X	X	
Constitutional symptoms	X	X	
Bone marrow fibrosis	X		
High-risk karyotype		X	X
Absence of good-risk CALR type 1 mutation	X	X	X
Presence of high-risk ASXL1 mutation	X	X	X
Presence of high-risk SRSF2 mutation	X	X	X
Presence of high-risk EZH2 mutation	X	X	
Presence of high-risk IDH1/IDH2 mutation	X	X	
Presence of U2AF1 mutation		X	X

^a^Defines sex-specific hemoglobin thresholds (severe: women < 8 g/dL and men < 9 g/dL, moderate: women 8–9.9 g/dL and men 9–10.9 g/dL)

Using the same patient cohort as the MIPSS+ V2.0, the GIPPS stratifies patients only by molecular and cytogenetic information into one of four risk categories. The cytogenetic and molecular risk categories are nearly identical, with both scoring systems delineating the favorable prognosis associated with type 1 CALR^ex9^ mutations compared with type 2, the unfavorable prognosis associated with *ASXL1*, *SRSF2*, and *U2AF1* abnormalities, and the poor survival seen with a complex karyotype. In the GIPPS multivariate analysis, *EZH2* and *IDH-1/2* mutations were not associated with inferior prognosis, a finding that will require additional validation.

Recently, a large exome-sequencing effort of 69 myeloid cancer genes in over 2000 patients with MPNs was reported, including 309 patients with MF^43^. In addition to the known poor prognostic influence of mutations in epigenetic and spliceosome components, the study identified a particularly poor-risk cohort with *TP53* mutations and a rare but significant association of *NRAS* mutations with MF. The authors designed a novel personalized MPN calculator incorporating clinical variables, cytogenetic abnormalities, as well as the ability to input individual mutations to predict OS and risk of progression to AML (https://cancer.sanger.ac.uk/mpn-multistage/). Although the model has yet to be prospectively validated, its novelty is in the paradigm of MPN subtype classification based on shared biology. With the calculator, one can input a hypothetical triple-negative 35-year-old male with MF and otherwise no risk factors (GIPPS intermediate-1 risk) and observe that the 5-year AML risk rate increases from 7.9% to 33.3% by the presence of a *TP53* mutation. It is these patients, young and clinically low risk, who are the key to identify early in their disease for closer monitoring and consideration of ASCT.

## Standard care in MF

### Low-risk and asymptomatic MF

Unfortunately, effective disease-modifying therapy for MF, outside of ASCT, is not a present reality in MF (Table 2)^44^. Thus, disease management is focused on the relief of symptoms and improvement in quality of life, with observation an acceptable choice for patients with asymptomatic low and intermediate-1 risk disease. A number of strategies can be considered for symptomatic low-risk patients tailored to the clinical situation. Interferon alfa, peg-interferon alfa, and hydroxyurea can produce meaningful reductions in spleen size and even reduce bone marrow fibrosis in some patients. Modest improvements in anemia secondary to MF are observed with erythropoietin-stimulating agents or androgens such as danazol^45^. The presence of the 5q− cytogenetic abnormality increases the likelihood of response to lenalidomide, which both itself and its parent compound thalidomide have been studied in combination with corticosteroids in MF.

**Table 2:** Standard of care in myelofibrosis

	Comments on clinical efficacy
**Low-risk, intermediate-1, asymptomatic disease**	
∙ Interferons (interferon alfa, peg-interferon)	Small number of patients will have improvement in bone marrow fibrosis
∙ Hydroxyurea	Useful for symptomatic splenomegaly
∙ IMIDs (thalidomide, lenalidamide)	IMIDs with increased efficacy in patients with 5q abnormality (lenalidomide).
∙ Erythropoietin-stimulating agents ∙ Androgens	Improvement in symptomatic anemia in selected patients
**Intermediate-2, high-risk, symptomatic disease**	
∙ JAK-2 inhibitors: Ruxolitinib, Fedratinib	Improvement in symptoms, splenomegaly. Longer follow up with ruxolitinib demonstrates possible overall survival benefit. Minority of patients with improved bone marrow fibrosis. Rare molecular remissions.
**Refractory, blast-phase disease**	
∙ Hypomethylating agents (azacitidine, decitabine)	Approximately 30% response rates, although can be durable
∙ Cytotoxic chemotherapy	Often last resort or after frank leukemic transformation, with poor response rates

Therapies are listed according to DIPSS risk assessment of disease, understanding that observation alone is also an appropriate strategy for low-risk patients. Patients with intermediate- and high-risk disease should also be evaluated for allogeneic stem cell transplant

### Ruxolitinib in symptomatic, intermediate-2, and high-risk MF

The discovery of JAK2^V617F^ as the most common molecular mutation in MPNs led to the development of ruxolitinib, a JAK1/2 inhibitor that is currently the only Food and Drug Administration (FDA)-approved JAK inhibitor in MF. The efficacy of ruxolitinib was confirmed in two randomized trials published in 2012, COMFORT 1 and 2, which both satisfied their primary endpoints of reduction in spleen size and symptomatic improvement in patients with intermediate-2 or high-risk MF^46–48^. Accepting the limitations of post-hoc analyses, combining data from both trials suggests an OS benefit, likely based on improvement in performance status, for patients exposed to ruxolitinib^49,50^. Additional follow-up has also shown ruxolitinib has some disease-modifying properties, as approximately one-third of patients showed improved bone marrow fibrosis at 5 years compared with 3% receiving best supportive care^51^. However, of 236 patients analyzed, only 6 cleared the JAK2^V617F^ mutation on therapy, suggesting molecular remissions are unlikely with single-agent ruxolitinib. Nonetheless, ruxolitinib therapy remains the standard option for symptomatic, intermediate-2, and high-risk MF with close monitoring for signs of disease progression, drug-related cytopenias, skin cancers, and opportunistic infections.

### Blast phase MF

Management of MF upon transformation to blast-phase is a clinical challenge with a historical survival rate of only several months^52^. Response rates of ~30% are achieved with single-agent use of the hypomethylating agents^53^. A phase II trial combining decitabine with ruxolitinib in 25 patients recently reported an overall response rate of 61%, albeit with a median OS still on the order of months^54^. However, the durability in patients with a response can be meaningful, as three patients were bridged to transplant on combination therapy. Induction chemotherapy regimens, similar to those used in AML, may be considered, but response rates are often poor^44^. With no clear standard of care, clinical trial participation should be encouraged.

## Expanding role for ASCT in MF

### Current guidelines for ASCT in MF

ASCT is the only therapy in MF that offers a chance for long-term remission or cure, but carries high treatment-related morbidity and mortality^55^. Currently, the NCCN Guidelines recommend that patients with DIPSS intermediate-2 or high-risk disease be considered for ASCT^44^. This suggestion is based on retrospective data in these risk groups showing approximately one-third are alive 5 years after transplant compared with <5% of high-risk patients treated with best available therapy^56,57^. DIPSS intermediate-1 and low-risk patients who underwent ASCT had lower 5- and 10-year survival rates compared with patients managed with conventional therapy; thus, transplant is not routinely performed in these cases. Various conditioning regimens have been used successfully, with myeloablative regimens favored based on patient tolerability. A number of studies have showed higher long-term survival rates with matched sibling donors compared with unrelated donor transplants, which is likely due to increased rates of graft-vs.-host disease in the later and not unique to MF. There does not appear to be a benefit of pretransplant splenectomy and this is not routinely recommended. It should be mentioned the ruxolitinib maintenance therapy has been investigated post-ASCT setting and appears well-tolerated, but larger patient cohorts are needed to confirm its efficacy^58^.

### Molecular-based transplant decisions in MF

A shortfall of the current guidelines for HSCT in MF is that a DIPSS risk assessment fails to incorporate the number of recently identified high-risk molecular mutations that have prognostic implications in MF. Furthermore, development of significant clinical complications that translate to higher DIPSS risk can decrease a patient’s candidacy or fitness for transplant. For this reason, recently modified prognostic scoring systems aim to identify transplant candidates by molecular/genetic risk (MIPSS+ V2.0, GIPPS), although this approach still needs prospective validation^6,59^. Using GIPPS, one readily identifies patients with very poor prognoses (GIPPS ≥ 3, 5-year survival of 14%) that should immediately be considered for ASCT and very good prognoses (GIPPS = 0, 5-year survival of 94%) that should be observed. The remaining patients (~70%) can then be reassessed with the MIPSS+ V2.0, which will result in a reclassification of high-risk or very-high risk in ~20% of patients. Ten-year OS in these MIPSS+ V2.0 patients is 10% and <2%, respectively, thus demonstrating the need for ASCT referral. A special consideration for ASCT can also be given to patients harboring *TP53* and *NRAS* mutations, which are associated with poor prognosis but not included in the current prognostic scoring systems^43^.

### Transplantation of elderly patients

In the past, ASCT was offered to MF patients under the age of 65 years; however, there have been recent reports of favorable outcomes in fit, older patients, reiterating that selection of patients should be on fitness and not age alone^60,61^. In a retrospective cohort with a median age of 67 years, Daghia et al.^61^ implemented a reduced intensity conditioning regimen prior to ASCT, which produced a 94% engraftment rate and limited the time to engraftment to 13 days. Over 90% of patients had intermediate-2 or high-risk DIPPS status and, impressively, a 64% 6-year estimated OS was observed, substantially better than would be historically expected in this population.

## Future therapies in MF

### Revisiting JAK2 inhibition: novel inhibitors and ruxolitinib combinations

**Fig. 2:**
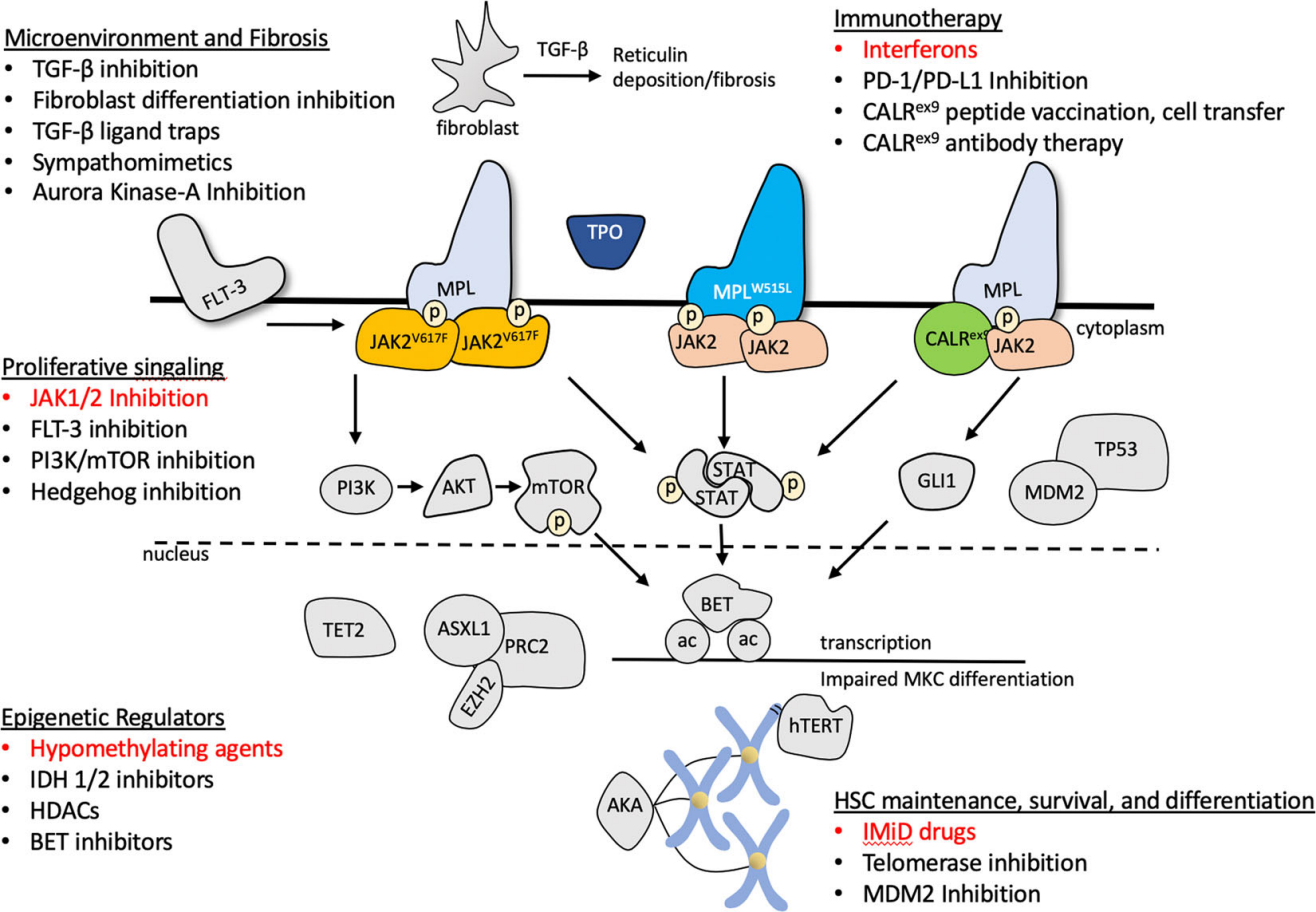
Therapeutic strategies for disease-modifying therapy myelofibrosis. These are classified as inhibitors of proliferative signaling, epigenetic regulators, agents involved in HSC maintenance, survival, and differentiation, immune therapies, and antifibrotic agents. Classes in red depict areas with FDA-approved agents (see Table 2). Signaling pathways cooperating with JAK-STAT activation include the tyrosine kinase receptor FLT3, the PI3K/mTOR axis, and Hedgehog signaling mediated by GLI1

Unfortunately, ruxolitinib is not sufficient in eliminating the underlying myeloid progenitor clone, as disease inevitably returns with therapy discontinuation. For this reason, and due to the increased rates of opportunistic infections on ruxolitinib therapy, many other strategies are being developed for treatment of MF (Fig. 2). This includes at least three other JAK kinase inhibitors: momelotinib, pacritinib, and fedratinib.

Momelotinib was prospectively compared with ruxolitinib in the SIMPLIFY-1 trial^62^ and after progression on ruxolitinib in the SIMPLIFY-2 trial^63^. Although less anemia and thrombocytopenia were observed with momelotinib, it showed less symptom control than ruxolitinib and no benefit for patients who progressed on ruxolitinib therapy. Pacritinib, a dual JAK/FLT3 inhibitor, has been investigated in two prospective randomized trials, PERSIST 1 and 2, which did not exclude patients with thrombocytopenia^64,65^. Activation of the FLT3 tyrosine kinase potentiates JAK/STAT signaling, thus providing an additional signaling target in MF^66,67^. In PERSIST 2, pacritinib was superior to the best available therapy, which included ruxolitinib, but had to be terminated early due to a clinical hold over concern for increased rates of bleeding and cardiac toxicity. This clinical hold has since been lifted and further development of pacritinib may offer a therapy option to patients with MF unable to tolerate ruxolitinib due to hematological toxicity. Fedratinib is a JAK-selective inhibitor that demonstrated efficacy in the JAKARTA-1 and -2 trails in treatment-naive and ruxolitinib refractory patients, respectively^68,69^. However, clinical development of the drug was halted due to neurological toxicity, specifically Wernicke’s encephalopathy. It has been determined that these events were unlikely related to fedratinib and the drug recently received FDA approval for intermediate-2 and high-risk MF patients. A number of clinical trials are now underway to investigate novel combinations with ruxolitinib for increased efficacy and potentially disease-modifying effects, and are listed for reference in Table 3.

**Table 3:** Ruxolitinib combinations currently under investigation

Name	Target	Study ID number
Therapies targeting abnormal epigenetic regulation		
Decitabine	Hypomethylating	NCT02076191
Azacitidine	Hypomethylating	NCT01787487
Panobinostat	HDAC	NCT01693601
Pevonedistat	HDAC	NCT03386214
Pacrinostat	HDAC	NCT02267278
CPI-0610	BET	NCT02158858
Therapies inhibiting coactivated JAK-signaling pathways		
Thalidomide	IMID	NCT03069326
Pomalidomide	IMID	NCT01644110
Lenalidomide	IMID	NCT01375140
Vismodegib	Hedgehog	NCT02593760
Sonidegib	Hedgehog	NCT02718300
TGR-1201	PI3K-delta	NCT02493530
INCB050465	PI3K-delta	NCT02718300
Idelalisib	P13K	NCT02436135
PIM447, LEE011	PIM kinase, CDK4/6	NCT02370706
SL-401	IL-3 signaling	NCT02268253
Other strategies		
Navitoclax	BCL-2 mimetic	NCT03222609
Peg-interferon Alpha-2a	Immune therapy	NCT02742324

Combinations are divided by mechanism of action, including therapies attempted to restore normal epigenetic regulation, inhibitors of proliferative and oncogenic signaling, and otherwise unclassified

### Reversing bone marrow fibrosis in MF

Another therapeutic avenue under investigation centers around the hypothesis that reversal of the altered bone marrow microenvironment and fibrosis will restore normal hematopoiesis in MF. The accumulation of atypical megakaryocytes may play a central role in this process through secretion of proinflammatory and profibrotic cytokines such as transforming growth factor (TGF)-β^70^. A pharmacological and genetic screen to identify regulators of megakaryocyte differentiation revealed that inhibition of Aurora kinase A (AURKA) promoted polyploidization and maturation of malignant megakaryocytes^71^. The Aurora kinases participate in chromosomal segregation during cell division and endomitosis, the unique cell cycle process that leads to the polyploidization of megakaryocytes^72^. An AURKA inhibitor MLN8237 (alisertib) was tested in the *Jak2*^*V617F*^ and *MPL*^*W515L*^ MPN models, and preferentially induced apoptosis and differentiation of mutant megakaryocytes, reduced TGF-β secretion, and improved bone marrow fibrosis^73^. Subsequently, alisertib was recently shown to have single-agent activity in patients with MF^74^.

Other strategies aimed at reversing bone marrow fibrosis include direct inhibition of TGF-β signaling with LY2157299 (galunisertib), a TGF-β receptor kinase inhibitor, which demonstrated significantly decreased bone marrow fibrosis in both JAK2^V617F^ and MPL^W515L^ mouse models^75^. Sotatercept and luspatercept are activin receptor type IIA ligand traps designed to sequester natural ligands to the TGF-β receptor and inhibit signaling. Both of these agents are currently in early-stage human trials for MF patients with anemia^76,77^. The human recombinant form of pentraxin-2, PRM-151, acts at sites of tissue injury to decrease myofibroblast differentiation, which contributes to progression of fibrosis^78^. PRM-151, alone and in combination with ruxolitinib, improved cytopenias and bone marrow fibrosis in a small cohort of patients with MF and post-MPN MF^79^.

### Immune therapies in MF

Given that ASCT offers the only curable option to MF patients, there is likely a “graft vs. MF” effect that eliminates early HSC precursors, as observed in other hematologic malignancies. This mechanism probably reflects the ability of recombinant interferon to reduce bone marrow cellularity and fibrosis as a single agent^80^, and has led to the testing of pegylated interferon with ruxolitinib^81^ (Table 3). In addition, there are currently three PD-1/PD-L1 checkpoint inhibitors durvalumab, nivolumab, and pembrolizumab, under evaluation in early phase I/II trials in patients with MF.

Perhaps the most promising rationale for immunotherapy is in patients with CALR^ex9^ disease. As all CALR^ex9^ mutations result in a 1 bp frameshift mutation, they share an identical altered C-terminal amino acid sequence that serves as a potential tumor antigen. It has been demonstrated that T-cells isolated from CALR^ex9^ patients recognize mutant CALR^ex9^ epitopes, and that CD4^+^ T-cells isolated from these patients are activated by autologous CALR^ex9^ HSCs^82,83^. This provides rationale for further development of CALR^ex9^-directed vaccines, immune cellular therapy, or neutralizing monoclonal antibodies.

### Other treatment considerations in MF

Tefferi et al.^84^ studied imetelstat, an oligonucleotide inhibitor of the human telomerase reverse transcriptase enzyme (hTERT), in 33 patients with MF. Telomeres are repetitive DNA sequences located at chromosome ends that protect DNA-coding regions and are maintained through hTERT activity. Telomeres shorten with age and repeated cellular division, and therefore it is hypothesized that the clonal drivers of MF, as well as other cancers, have heightened sensitivity to hTERT inhibition due to an increased need to maintain telomeres. This has been confirmed in vitro as imetelstat preferentially induces apoptosis in MF progenitor cells, allowing repopulation of the bone marrow with normal HSCs^85^. In the initial phase I trial, imetelstat induced a response in seven patients, including four with complete response and reduction in bone marrow fibrosis. A subsequent phase II dose randomization trial that was recently presented at the American Society of Hematology annual meeting found that 9.4 mg/kg imetelstat given every 3 weeks resulted in a 10.2% spleen response and 32% symptom response^86^. OS of these patients also appeared longer than historical controls. Larger studies are needed to validate this observation, as well as design dosing schedules that minimize myelosuppression.

There is increasing evidence that an altered bone marrow microenviroment, in addition to the mutated HSCs, play a role in progression in MPNs/MF. Specifically, nestin-positive bone marrow mesenchymal stem cells (nMSCs) innervated by sympathetic nerve fibers are consistently reduced in patients with MPNs^87^. Administration of the sympathomimetic drug mirabegron restored nMSCs and reduced reticulin fibrosis in a phase II study^88^. What is particularly novel about this finding is that JAK2^V617F^ allele burden is not decreased with mirabegron, suggesting activated STAT signaling and progression of fibrosis may occur independently.

A possible explanation for the modest disease-altering effects seen with ruxolitinib monotherapy is the failure to eliminate the clonal HSCs driving the disease. These cells appear to be protected from apoptosis through upregulation of MDM2, the master negative regulator of TP53^89^. An open-label phase 2 trial with the MDM2 inhibitor KRT-232 is currently enrolling for patients who failed ruxolitinib therapy (NCT03662126).

Over the past year, a number of new agents have become available in the management of AML^90^. It will be important to test their efficacy in MF, particularly in the blast phase where traditional chemotherapy regimens are often unsuccessful. Specifically, although <5% of patients with MF harbor an *IDH1/2* mutation, it’s intuitive to test whether the FDA-approved IDH inhibitors enasidinib and ivosidenib provide benefit. Other agents with efficacy in AML, but remaining untested in MF, include CPX-351 (liposomal daunorubicin and cytarabine), gemtuzumab ozogamicin (anti-CD33 drug conjugate), and enhancers of apoptosis (navitoclax, venetoclax).

## Conclusions

Since the discovery of JAK2^V617F^ mutations in MPNs over 10 years ago, significant advancements have been made in understanding the biology of MF. Although activated JAK-STAT signaling is a hallmark abnormality in MF, even in cases of triple-negative MF, *JAK2*, *MPL*, and *CALR* mutations are often accumulated within already abnormal HSC clones. This is one possible reason why JAK inhibition with ruxolitinib is not sufficient in producing long-term disease remissions and reversal of bone marrow fibrosis. Therefore, efforts are underway to better understand the initiating events in MF to identify novel targets that alone and in combination with JAK inhibition have disease-modifying properties. As the knowledge of the molecular abnormalities in MF expands, there will be improvements in MF risk models as well, integrating both clinical and genetic information that impact prognosis. Intriguing molecularly based therapies include the use of IDH inhibitors, spliceosome inhibitors, and immunotherapeutic approaches particularly for patients with *CALR*-mutated MPNs.
